# Primary Vasculitis in Childhood: GPA and MPA in Childhood

**DOI:** 10.3389/fped.2018.00226

**Published:** 2018-08-16

**Authors:** Mehul P. Jariwala, Ronald M. Laxer

**Affiliations:** ^1^Saskatoon Health Region, Saskatoon, SK, Canada; ^2^Department of Pediatrics, Royal University Hospital, University of Saskatchewan, Saskatoon, SK, Canada; ^3^The Hospital for Sick Children, University of Toronto, Toronto, ON, Canada

**Keywords:** childhood vasculitis, microscopic polyangiitis, granulomatosis with polyangiitis, ANCA—associated vasculitis, small vessel vasculitis

## Abstract

Childhood onset anti-neutrophilic cytoplasmic antibody (ANCA) associated vasculitis (AAV) is a rare group of primary systemic vasculitides affecting medium and small blood vessels. AAV includes granulomatosis with polyangiitis (GPA), microscopic polyangiitis (MPA), eosinophilic granulomatosis with polyangiitis (EGPA), and renal limited ANCA vasculitis. These disorders are associated with severe clinical manifestations, frequent relapses and a high cumulative morbidity, and often present with multisystem involvement. Renal involvement is common in the pediatric age group, characterized by pauci-immune necrotizing and crescentic glomerulonephritis which frequently progresses to chronic kidney disease in adulthood. ANCAs against proteinase 3 (PR3-ANCA) or myeloperoxidase (MPO) (MPO-ANCA) remain the hallmark of AAV and are integral to the disease pathogenesis. Newer understanding of neutrophil extracellular traps and complement activation have provided better insights into disease pathogenesis. A pediatric vasculitis working group has developed and validated childhood vasculitis classification criteria and disease activity and damage scores. No specific pediatric treatment recommendations exist due to rare nature of the illness in pediatric population. Smaller case series have been published on the efficacy of adult treatment regimens in pediatric patients. The prognosis often remains guarded with frequent relapses and a high cumulative morbidity. The aim of this article is to provide a comprehensive review on pediatric AAV with a focus on recent observations regarding epidemiology, disease pathogenesis, treatment, and prognosis.

## Introduction

Anti-neutrophilic cytoplasmic antibody (ANCA) associated vasculitides (AAVs) are primary systemic vasculitides characterized by necrotizing arteritis with few or no immune deposits in small to medium-sized arteries in multiple organs. This group comprises granulomatosis with polyangiitis (GPA), microscopic polyangiitis (MPA), eosinophilic granulomatosis with polyangiitis (EGPA), and renal limited ANCA vasculitis (1). These diseases often have the presence of circulating autoantibodies (ANCA) that are usually directed against myeloperoxidase (MPO) or proteinase 3 (PR3) antigens. In the pediatric population, GPA is more common than MPA and EGPA. Renal involvement in AAV is characterized by rapidly progressive pauci-immune necrotizing and crescentic glomerulonephritis contributing significantly to the morbidity and progression to end stage renal disease. Treatment is often extrapolated from adult studies due to rare nature of this illness in the pediatric population. Early recognition and treatment remains pivotal to the better outcome in these patients. This review focuses on recent publications on epidemiology, update on AAV pathogenesis, recently described pediatric cohorts, disease outcome measures and the Canadian Vasculitis research network (CanVasc) endorsed pediatric treatment guidelines.

## Epidemiology

AAV is much more common in adults compared to the pediatric population. GPA is the most frequent, followed by MPA and EGPA. The peak incidence of GPA is in fourth-fifth decade of life and is more common in males. Multiple adult studies have been published on the epidemiology of AAV from Europe, Japan, the USA, New Zealand and Australia. Europe reports overall incidence rates of AAV from 13 to 20 per million (2).

Epidemiological data in childhood AAV are scarce. The French registry reported an increasing annual incidence of AAV over the 25-year period from 0.10 in 1986–90 to 0.45 per million children from 2006 to 2010 (3). The reported annual incidence rate per million children from a Swedish study was 1.4 (4). In contrast to this, the reported incidence in Southern Alberta, Canada continues to increase from 2.75 cases/million/year in the last 15 years to 6.39 cases/million/year in the last 5 years (5). Childhood AAV has a higher female preponderance with a peak age at onset in second decade and median age at diagnosis of approximately 12–14 years (3, 5, 6).

## Pathogenesis

The precise etiopathogenesis of AAV is not fully elucidated. There appears to be a complex interplay of genetic susceptibility factors, environmental triggers and dysregulation in both the innate and adaptive immune systems contributing to the development of AAV. Multiple theories have been proposed to identify the pathogenic pathways in AAV.

Role of ANCA: ANCA have a central role in pathogenesis of AAV. The presence of ANCA indicates the involvement of the neutrophil as the effector cell. Higher levels of ANCA target antigens myeloperoxidase (MPO) or proteinase 3 (PR3) are noted on the surface of circulating neutrophils in AAV, which could be secondary to disturbed epigenetic modification (7, 8). *In vitro* studies have also demonstrated IgG-ANCA capable of inducing an oxidative burst releasing toxic oxygen radicals, primary granule release and surface activation in cytokine primed neutrophils with IgG-ANCA (9). This process eventually leads to endothelial damage and activation of the alternate complement pathway (10). The development of ANCA may result from a breakdown of tolerance.

### Mechanisms of tolerance breakdown

**Figure 1:**
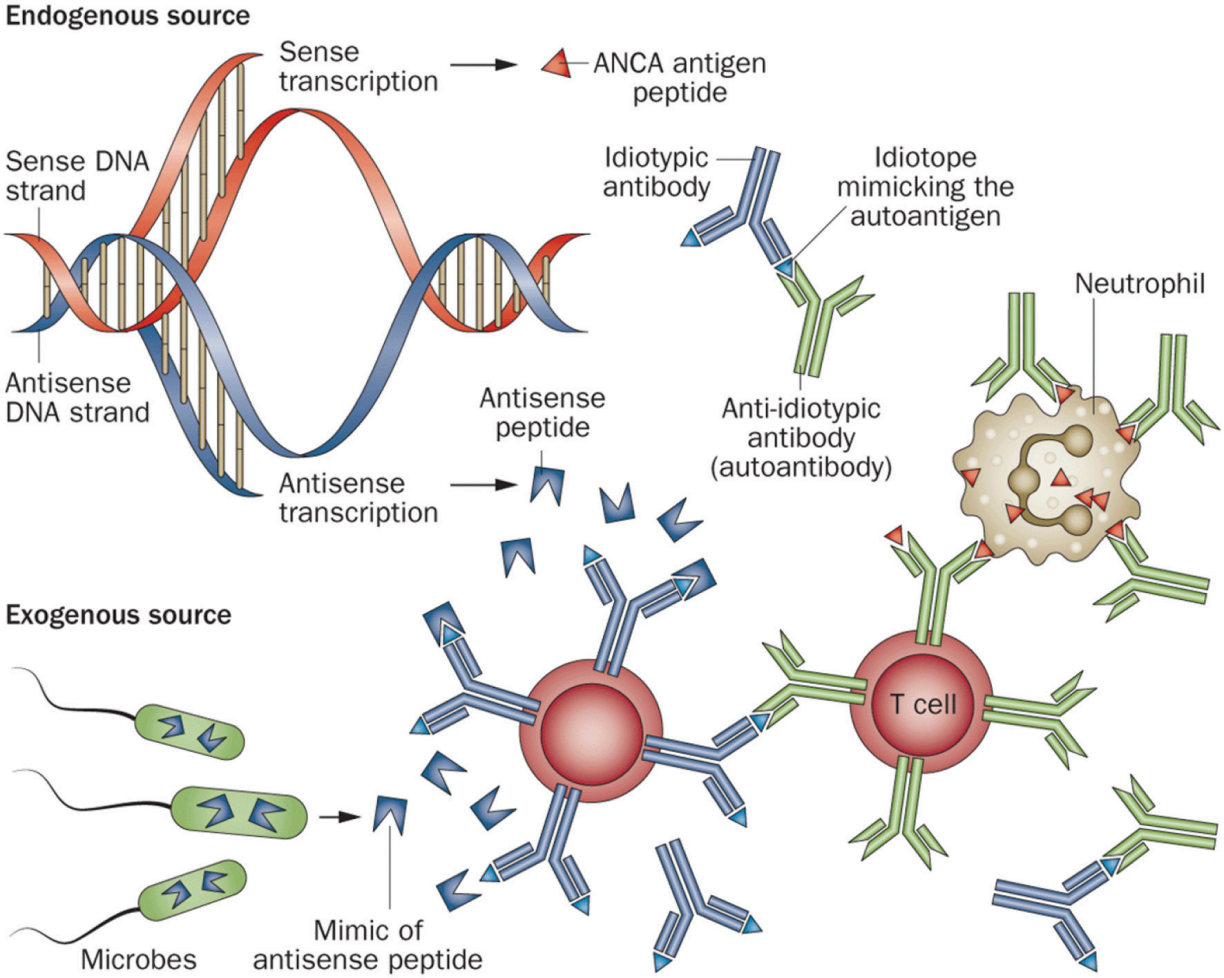
Diagram of the induction of an ANCA-mediated autoimmune response by an initial immune response to a peptide that is complementary to an autoantigen peptide. This complementary peptide immunogen could arise from antisense transcription of the antisense strand of the autoantigen gene, or could be a mimic of an antisense peptide that is produced by a symbiotic or pathogenic microbe. The anti-complementary peptide antibody idiotopes would engender an anti-idiotypic antibody response that cross reacts with the autoantigen epitopes that are complementary to the initial immunogenic peptide. Reprinted by permission from Springer Nature Terms and Conditions for RightsLink Permissions Springer Customer Service Centre GmbH: Nature. Jennette and Falk (11).

- Complementary Peptide Model: This theory hypothesizes that the initial immune response is to a peptide with complementary structure relative to the autoantigen. In AAV, these complementary peptides are derived from antisense transcription of the antisense strand of the autoantigen at the PRTN3 (the gene encoding PR3) or MPO loci. Alternatively, the complementary peptide can be a mimic of an antisense peptide that is produced by a symbiotic or pathogenic microbe. These can stimulate a B cell adaptive immune response leading to anti-idiotype antibodies which cross react with the autoantigen epitopes (Figure 1) (11).
- Molecular mimicry models: An infectious link to autoimmunity is well known in AAV. Chronic nasal carriage of Staphylococcus aureus has been identified as an independent risk factor in relapse of GPA (12). Kain and colleagues proposed a model of molecular mimicry wherein rats injected with gram-negative bacillus adhesion protein FimH developed pauci-immune focal necrotizing glomerulonephritis. Autoantibodies to human LAMP-2 are highly prevalent in pauci-immune FNGN. These antibodies share considerable homology to FimH and could induce antibodies to human LAMP-2 and initiate pauci-immune FNGN (13).

NETosis: Traditionally it was hypothesized that neutrophils die in small vessels by necrosis. However neutrophil extracellular traps (NETs) have been identified at the site of the vasculitic lesion (14, 15). NETosis is a type of programed cell death mechanism in which the neutrophils have the ability to extrude their DNA and proinflammatory bactericidal molecules creating NET-like structures. Patients with AAV possess elevated levels of NETs in the circulation (16, 17). NETs can lead to vascular necrosis, endothelial damage, expose immune-stimulatory molecules and can activate alternate complement pathways (18–20). Kessenbrock and colleagues showed that ANCA-stimulated neutrophils are capable of inducing NETs which contain proteinase-3 (PR3) and myeloperoxidase (MPO). This complex promotes the autoimmune response against neutrophil components in individuals with vasculitis (14).

Role of Apoptosis: Apoptosis (programmed cell death) is a vital component of the immune system, promoting resolution of inflammation by clearance of cellular debris by macrophages (21). In patients with GPA, spontaneous apoptosis of neutrophils is significantly less as compared to normal individuals. Neutrophils in these patients express higher membrane bound PR3 without degranulation as compared to healthy controls. The membrane associated PR3 on apoptotic neutrophil delays their clearance by macrophage and also act as a danger signal through the IL-1R1/MyD88 signaling pathway in a NO-dependent manner triggering a proinflammatory response by macrophages recruiting more neutrophils and monocytes (22, 23).

Role of complement pathway: The role of alternate complement pathway (ACP) activation was first described by Xiao et al. (24) who showed that complement component depletion in mice could prevent crescentic glomerulonephritis. Further animal studies by the same group suggested that knock-out mice for C5 and factor B after receiving anti-MPO IgG did not develop disease. These data suggest that ACP and the activation of complement C5 are critical to AAV pathogenesis. The activation of alternate pathway leads to increase anaphylatoxin C3a and C5a resulting in an inflammatory amplification loop which mediates the severe necrotizing inflammation of ANCA disease (24).

## Classification

The 1990 American College of Rheumatology (ACR) classification criteria were developed for GPA and EGPA and were derived from adult patient data (25). A new set of classification criteria was proposed in 2006 by European League against Rheumatism/Pediatric Rheumatology European Society (EULAR/PReS) group that included components of the 1990 ACR criteria along with common pediatric manifestations and inclusion of ANCA (26). These criteria were endorsed by EULAR, the Pediatric Rheumatology European Society, and Pediatric Rheumatology International Trial Organization (EULAR/PRINTO/PReS) and were published in 2010 (27). They included CT scan results, ANCA positivity and better descriptive terms for upper and lower respiratory involvement with specific mention of subglottic stenosis which were lacking in ACR criteria. In the ARChiVe cohort, a pediatric cohort of patients with AAV, the EULAR/PRINTO/PRES criteria were found to be more sensitive than the ACR criteria in classifying pediatric GPA (28). The EMA (European Medicine Agency) is a classification algorithm (29) which comprises of ACR criteria, Chapel Hill Consensus Conference (CHCC) definition, Lanham criteria for Churg Strauss syndrome (30) and presence or absence of ANCA. The EMA algorithm was reported as the most sensitive in diagnosing childhood GPA (28).

MPA was defined by Chapel Hill Consensus Conference (CHCC) in the small vessel vasculitis category as: “necrotizing vasculitis, with few or no immune deposits, predominantly affecting small vessels (i.e., capillaries, venules, or arterioles). Necrotizing arteritis involving small and medium arteries may be present. Necrotizing glomerulonephritis is very common. Pulmonary capillaritis often occurs. Granulomatous inflammation is absent” (1). No specific pediatric classification criteria for MPA were endorsed in the EULAR/PReS meeting in 2008. It was proposed more of a clinical syndrome in 2006 by EULAR/PReS group. The only modification to the Chapel Hill report was addition of ANCA to the description of microscopic polyangiitis.

The newly endorsed pediatric vasculitis classification criteria have several limitations such as no pediatric specific MPA criteria were defined, certain degrees of overlap between GPA and MPA have been noted and limited forms of the disease were not described in these criteria.

The criteria for classifying GPA according to the EULAR/PRINTO/PReS criteria are listed in Table 1.

**Table 1:** Final EULAR/PRINTO/PRES childhood GPA criteria.

**Criteria**	**Description**	**Sensitivity (%)**	**Specificity (%)**
**A PATIENT IS SAID TO HAVE GPA WHEN THREE OF THE FOLLOWING SIX CRITERIA ARE PRESENT:**			
Histopathology	Granulomatous inflammation within the wall of an artery or in the perivascular or extravascular area	54	99.6
Upper airway involvement	Chronic purulent or bloody nasal discharge or recurrent epistaxis/crusts/ granulomata Nasal septum perforation or saddle nose deformity Chronic or recurrent sinus inflammation	83	99
Laryngo-tracheo-bronchial involvement	Subglottic, tracheal, or bronchial stenosis	22	99.8
Pulmonary involvement	Chest x-ray or CT showing the presence of nodules, cavities or fixed infiltrates	78	92
ANCA	ANCA positivity by immunofluorescence or by ELISA (MPO/p or PR3/c ANCA)	93	90
Renal	Proteinuria >0.3 g/24 h or >30 mmol/mg of urine albumin/creatinine ratio on a spot morning sample Hematuria or red blood cell casts: >5 red blood cells/high power field or red blood cells casts in the urinary sediment or ≥2+ on dipstick Necrotizing pauci-immune glomerulonephritis	93.2	99.2

## Clinical features of AAV

### GPA

GPA is a systemic pauci-immune necrotizing small and medium-size vessel vasculitis associated with granulomatous inflammation (26). The classic form of GPA presents with a triad of upper and lower respiratory tract involvement with renal manifestation presenting as pauci-immune crescentic glomerulonephritis (GN). Disease manifestations in GPA have been described in the larger cohorts listed in Table 2.

**Table 2:** Clinical features of GPA at presentation in the largest pediatric cohorts reported.

	**Cabral et al. (ARChiVe) (6)**	**Bohm et al. (31)**	**Sacri et al. (3)**	**James et al. (33)**	**Calatroni et al. (37)**	**Akikusa et al. (36)**
Type of study	Retrospective and Prospective	Retrospective	Retrospective	Retrospective	–	Retrospective
No of patients	183	56	28	28	31	25
M/F	70/113	18/38	7/21	68%	11/22	5/20
Median age at diagnosis	12	–	12.8	14.7	14	14.5
Ethnicity	Predominant Caucasians (59%), unknown (44%)	Caucasians	Caucasians	Caucasians (75%) and Asian (21%)	Caucasian	Caucasian
Classification used	MD diagnosis	EULAR/ PRINTO/ PRES	EULAR/ PRINTO/ PRES	EULAR/ PRINTO/ PRES or ACR1990	Unclear	ACR Criteria
**CLINICAL FEATURES (%)**						
Constitutional symptoms (fever, malaise and weight loss)	88	89	82	79	68	96
Upper respiratory involvement (ear, nose, throat)	70	91	75	93	78	84
Lower respiratory tract involvement (hemoptysis, nodules, pulmonary hemorrhage and pulmonary infiltrates)	74	78.5	68	82	52	80
Renal (elevated creatinine, biopsy proven GN, abnormal urinalysis)	83	68	78.5	71	65	88
Ocular (scleritis, conjunctivitis)	43	34	21.4	21	45	52
Gastrointestinal	36	16	17.8	29	6	12
Musculoskeletal (arthralgia, myalgia, arthritis)	14	59	57.1	61	45	96
Mucocutaneous	47	64	53.5	33	26	32
Nervous system (headache)	20	14	3.5	4	6	8
Cardiovascular (venous thrombosis)	5	0	0	11	6	20

**Figure 2:**
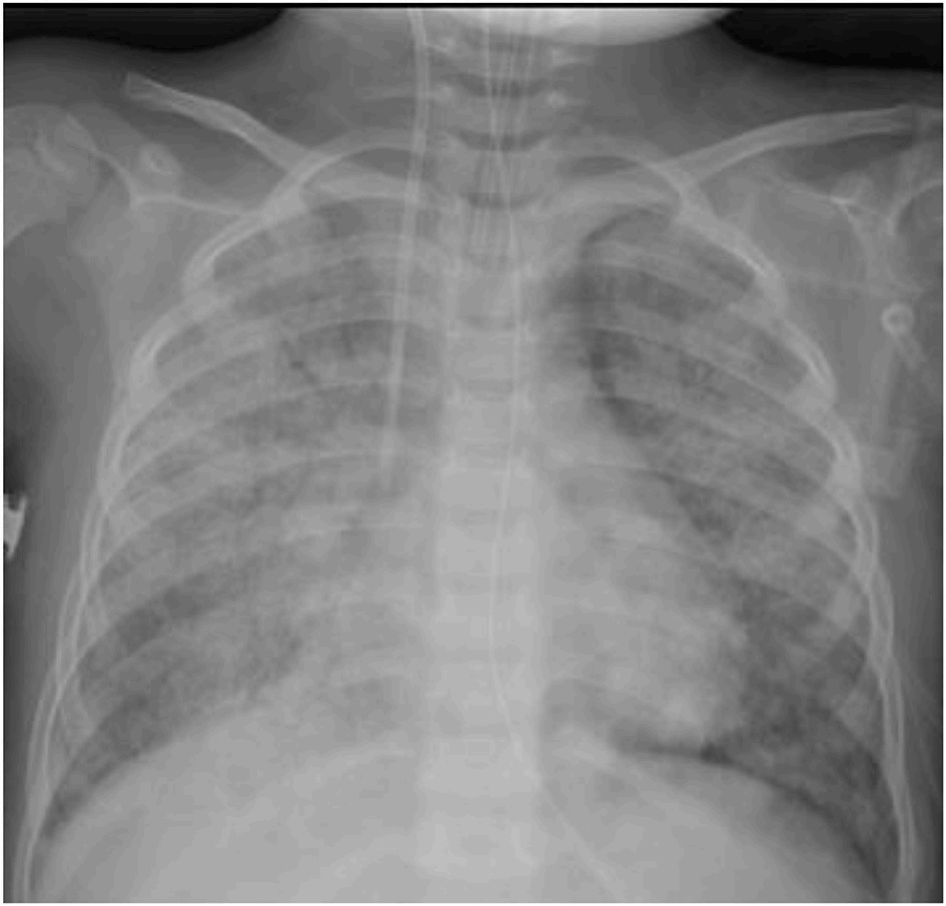
Diffuse pulmonary hemorrhage in 3-year-old presenting with pulmonary-renal syndrome diagnosed as MPA.

Involvement of the upper respiratory tract remains the most feature in children with GPA. Childhood GPA presents either as localized granulomatous disease with a chronic course or as an acute small vessel vasculitis characterized by pulmonary hemorrhage and/or rapidly progressive renal involvement. The majority of patients present with symptoms of upper or lower respiratory tract including epistaxis, sinusitis, otitis media, and hearing loss. Subglottic stenosis; one of the severe complication of GPA was reported in at presentation from 10 to 20% (6, 31). It is also more common in pediatric as compared to adult cohorts and hence proposed in pediatric classification criteria (26, 32). Alvelolar, pleural or bronchial tissues can be involved as a part of lower respiratory tract involvement, presenting with cough, wheezing, hemoptysis, bronchial stenosis, or a catastrophic pulmonary hemorrhage. Imaging findings of nodules, fixed pulmonary infiltrates and cavitation were noted in patients ranging from 21 to 80% (6, 33) (Figure 2).

### MPA

Upper respiratory tract involvement is rarely noted in MPA. In fact, per the case definitions, involvement of upper respiratory tract often precludes the diagnosis of MPA. Lower respiratory tract involvement is common with MPA often presenting as hemoptysis, anemia secondary to chronic, low-grade pulmonary hemorrhage with pulmonary hemosiderosis, or as catastrophic pulmonary hemorrhage noted in up to 42% patients (6). Granulomatous inflammation does not occur in MPA. Pulmonary-renal syndrome is one of the most severe AAV manifestations and can often reveal the disease or occur during its evolution. Renal involvement is very common and noted in 94–100% of the patients (3, 6, 34, 35). Disease manifestations in MPA have been described in the larger cohorts listed in Table 4.

### Clinical features common to both GPA and MPA

Pauci-immune necrotizing GN is a severe manifestation of GPA and MPA often leading into renal failure contributing to significant morbidity. Renal involvement is noted in up to 80% of patients with GPA. The clinical features of renal involvement include hypertension, edema, proteinuria, and hematuria. The largest cohort, published by Cabral et al. reported elevated creatinine, requirement of dialysis and end stage renal disease in 60, 25, and 10% respectively (6). Histopathological findings consistent with pauci-immune GN and/or necrotizing glomerulonephritis were noted in 94% of the patients and biopsy findings of vasculitis in 29% (6).

**Figure 3:**
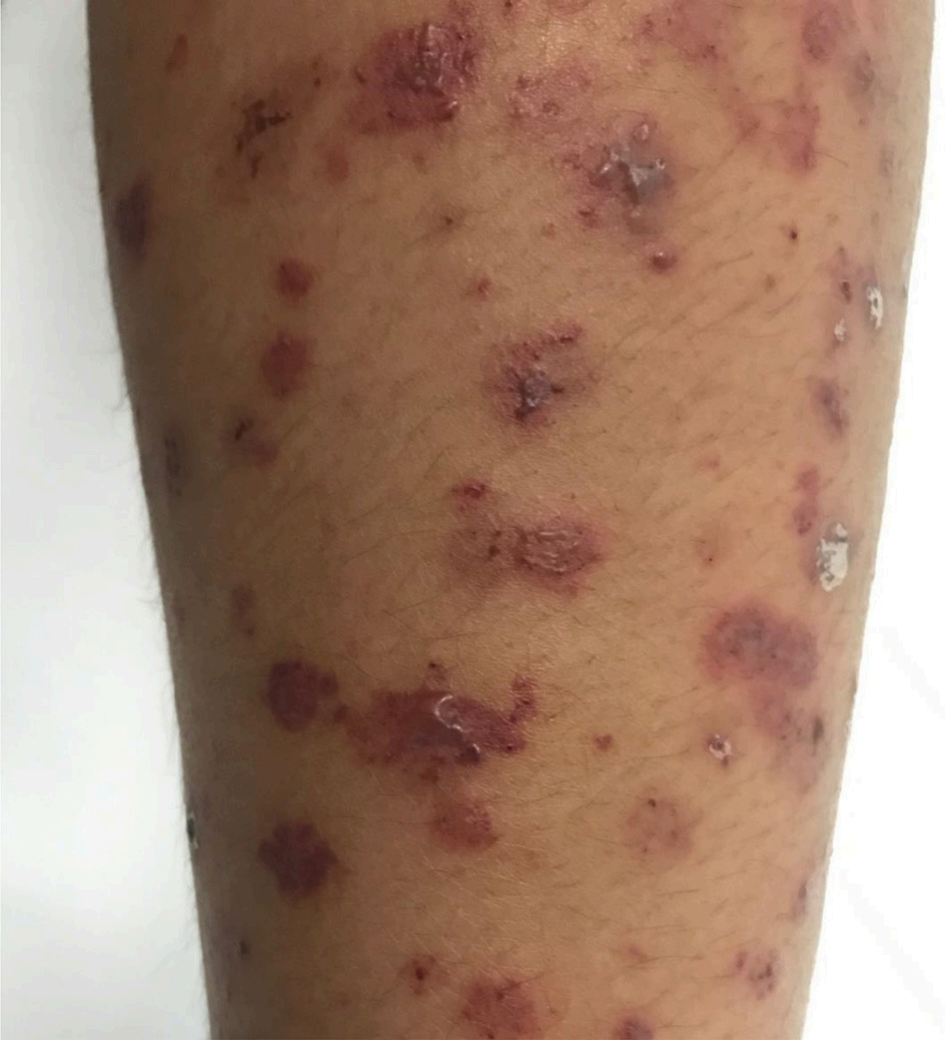
Eight year old diagnosed with GPA with leucocytoclastic vasculitis involving upper and lower limbs.

More than 80% of patients across all cohorts reported constitutional symptoms of malaise, weight loss and/or fever. The recently published GPA cohorts report higher incidence of gastrointestinal (GI) manifestation, in 30–36% of patients (6, 33). Common GI manifestations in AAV include chronic nausea, diarrhea, and non-specific abdominal pain. Cabral et al. reported <5% of children with bloody diarrhea or ischemic abdominal pain (6). Mucocutaenous manifestation include oral and genital ulcers, palpable purpura (Figure 3), petechial rash, livedo, and subcutaneous nodules. Eye involvement was reported in up to 20–50% across the different cohorts. Common eye symptoms in order of frequency were conjunctivitis, episcleritis, proptosis secondary to retro-orbital mass, and keratitis. Arthritis, arthralgia, myalgia, and muscle weakness were the common MSK symptoms in both MPA and GPA. Neurologic symptoms were less common across all cohorts and included non-specific findings of headache and dizziness. The highest incidence of venous thrombosis (20%) was reported in GPA by Akikusa et al. but remained less common in other cohorts (36). Cardiovascular involvement in GPA was more common than MPA (Table 3).

**Table 3:** Clinical differences between GPA and MPA.

	**GPA**	**MPA**
Constitutional symptoms	Common	Common
ENT disease	Common and often severe Sinusitis, epistaxis, otitis media, septal perforation, subglottic stenosis	Very mild to absent
Pulmonary involvement	Nodules, cavitatary lesions, fixed infiltrates, and pulmonary hemorrhage	Pulmonary hemorrhage
Ocular	Scleritis, episcleritis, orbital pseudotumor, uveitis, conjunctivitis	Episcleritis and conjunctivitis. Rare orbital pseudotumor
Kidney	Segmental pauci-immune necrotizing and crescentic glomerulonephritis (rare granulomatous lesions)	Segmental pauci-immune necrotizing and crescentic glomerulonephritis
Nervous system	Headache, peripheral neuropathy (rare)	peripheral neuropathy (common)
Cardiovascular	Venous thrombosis, Occasionally valvular lesions	Rare involvement.

**Table 4:** Clinical features of MPA in different cohorts at presentation in the largest pediatric cohorts reported.

	**Sacri et al. (3)**	**Cabral et al. (ARChiVe) (6)**	**Sun et al. (35)**	**Yu et al. (34)**
Type of study	Retrospective	Retrospective and Prospective	Retrospective	Retrospective
No of patients	38	48	20	19
M/F	4/34	13/35	4/16	1/18
Median age at diagnosis	14.2	14	NA	NA
Ethnicity	Caucasian (68%)	Caucasian (42%), Hispanics (13%)	NA	NA
Classification used	Watts et al (29)	EMA algorithm (6)	Revised Chapel- Hill CC (1)	Revised Chapel- Hill CC
**CLINICAL FEATURES (%)**				
Constitutional symptoms (fever, malaise, and weight loss)	78	85	50	63
Upper respiratory involvement (ear, nose, throat)	0	0	0	10.5
Lower respiratory tract involvement (hemoptysis, nodules, pulmonary hemorrhage and pulmonary infiltrates)	30	44	15	52.6
Renal (elevated creatinine, biopsy proven GN, abnormal urinalaysis)	94.7	75	100	100
Ocular (scleritis, conjunctivitis)	7.8	31	5	5.2
Gastrointestinal	10.5	58	15	9
Musculoskeletal (arthralgia, myalgia, arthritis)	29	52	10	0
Mucocutaneous	34.2	52	15	31.5
Nervous system (headache)	5.2	21	15	
Cardiovascular	0	6	0	0

## Disease activity scoring

The development of a validated scoring tool to measure disease activity, damage and outcome is crucial for pediatric vasculitis related clinical trials. The Birmingham Vasculits Activity Score (BVAS) is a validated multisystem disease activity tool assessment for primary systemic vasculitis in adults. The latest revision of BVAS is v.3, which was applied to the ARChiVe cohort and showed only weak to moderate correlations with PGA, ESR and treatment decision (38). Dolazelova et al. in 2012 published the Pediatric Vasculitis Activity Score (PVAS) by redefining the BVAS components and adding eight pediatric items in cutaneous, cardiovascular and abdominal sections (39). PVAS has 64 items in nine categories with higher score indicating higher disease activity. PVAS was validated in pediatrics patients with systemic vasculitis and can be used as to define disease activity for clinical trials and future research.

The Vasculitis Damage Index (VDI) is a standardized clinical assessment tool of damage in adult systemic vasculitides (40). There is no validated tool to assess disease damage in children with vasculitis. Pediatric modification of the Vasculitis Damage Index (PVDI) was proposed by Dolezalova et al. in 2014 (41). PVDI contains 72 items in 10 systems. Once validated, PVDI should serve as an important step toward better disease assessment in clinical trials in children with systemic vasculitis.

## Treatment

No specific pediatric management guidelines are available to guide the therapeutic approach in pediatric patients with AAV. Therefore, treatment recommendations are adapted from the adult clinical trials and expert consensus (42, 43). Survival rates in AAV has improved secondary to better disease management, the expertise of care teams at academic and referral vasculitis centers and treatment based on intensive remission induction followed by maintenance therapy. The Canadian Vasculitis research network (CanVasc) recommends that children with newly diagnosed AAV should be treated as per adult recommendations for induction of remission and then maintenance (43). Glucocorticoids and cyclophosphamide has been associated with dramatic improvement in patients with AAV, however this combination has not prevented relapses in the majority of patients and is associated with short and long term toxicity risk (44).

### Remission induction for newly diagnosed disease

The EULAR/ERA-EDTA (European League Against Rheumatism/ European Renal Association—European Dialysis and Transplant Association) and CanVasc recommend treatment with a combination of glucocorticoids and either cyclophosphamide (CYC) or rituximab (RTX) (42, 43). RTX is preferred as a first line remission induction therapy for patients in whom CYC is contraindicated or presents a risk of infertility. CYC can be administered either orally or as pulse intravenous dose (3–6 months) but the latter is preferred as it is associated with less cumulative dose and reduced risk of bladder-related complications. However, daily oral low-dose CYC is associated with a slightly lower rate of relapse on long-term follow up (45).

RTX was shown to be non-inferior to CYC at inducing remission in adults with organ or life-threatening disease (46, 47). RTX is usually administered as 4 weekly infusions of 375 mg/m2 as recommended in the RAVE (Rituximab in ANCA- Associated Vasculitis) (46) and RITUXVAS (Rituximab versus Cyclophosphamide in ANCA-Associated Renal Vasculitis) trials (47). RTX is being increasingly used in children with AAV as a first line induction therapy compared to CYC (48) because of reduced toxicity.

Glucocorticoids (GC) remain an important therapy in remission induction and maintenance. No RCTs have been published comparing different GC dosing. In life-threatening disease or those with major organ involvement, pulsed IV methylprednisolone 0.5–1 g/day for 3 consecutive days is recommended. Most adult guidelines recommend initial GC dose of 1 mg/kg/day and tapering to a desirable level of reaching a target GC dose of 10–12.5 mg by 3–5 month. In children (<15 years old), the initial dose of oral prednisone used is 1–2 mg/kg/day with a maximum of 60 mg/day (43). Daily calcium (500–1000 mg) and vitamin D (1000 IU) supplementation is recommended.

### Remission-induction of limited or non-severe non-organ-threatening AAV

Methotrexate (MTX) is recommended in combination with GC in those with limited or non-organ threatening AAV. In the NORAM trial (Non-renal Wegener's Granulomatosis Treated Alternatively with Methotrexate [MTX]), MTX was reported non-inferior to oral CYC in achieving remission induction but subsequent reports indicated less effective disease control than CYC-based induction therapy (49, 50). Mycophenolate mofetil (MMF) was compared to IV CYC in non-severe GPA in MYCYC (mycophenolate mofetil versus cyclophosphamide for remission induction of ANCA-associated vasculitis) trial. MMF was noted to be non-inferior to CYC but was associated with a higher rate of relapse (51).

#### Remission maintenance therapy

The CanVasc as well as EULAR/ERA-EDTA recommends remission maintenance treatment with a combination of low-dose glucocorticoids and either azathioprine (AZA), rituximab, methotrexate or mycophenolate mofetil. This therapy for AAV be continued for at least 24 months following induction of sustained remission.

In the Cyclophosphamide versus Azathioprine for Early Remission Phase of Vasculitis (CYCAZAREM) trial, AZA was shown to be equally efficacious as continuous CYC as maintenance treatment. This regime was also associated with fewer side-effects (52). MTX was shown to be well tolerated and effective in maintaining remission after induction with CYC and was proven to be of comparable efficacy to AZA (53, 54). Leflunomide, though associated with increased frequency of adverse events, was found to be more effective than methotrexate in remission maintenance at a dose of 30 mg/day (55). Leflunomide can be used as an alternative agent in patients with intolerance to MTX and AZA. In the International Mycophenolate Mofetil Protocol to Reduce Outbreaks of Vasculitides (IMPROVE) trial, MMF was shown to be less effective than azathioprine for maintaining disease remission (56).

The role of RTX in maintenance therapy has been investigated in patients with AAV after inducing remission either with CYC or RTX. It is considered a safe and effective alternative to AZA. The MAINRITSAN (Efficacy Study of Two Treatments in the Remission of Vasculitis) was the first randomized trial comparing RTX (500 mg at remission, at 2 weeks and then once every 6 months till 18 months) to daily AZA (which was tapered after 12 months). Patients receiving RTX had sustained remission compared to AZA without significant adverse events (57). MAINRITSAN 2 (https://clinicaltrials.gov/ct2/show/NCT01731561) explores the RTX maintenance treatment based on ANCA estimation and CD19 lymphocytes. The RITAZAREM trial is planned to evaluate two remission-maintenance strategies of repeated doses of RTX compared to daily orally administered AZA for 24 months following induction with RTX (58).

#### Relapsing disease

Both the CanVasc and EULAR/ERA-EDTA guidelines recommend switching from RTX to CYC and vice versa for relapsing AAV. In those who continue to have persistent active disease, intravenous immunoglobulin may be used as an adjunctive therapy.

#### Role of plasmapheresis (PLEX)

The role of PLEX in AAV is not well defined. It is recommended to be used for rapidly progressive glomerulonephritis in the setting of new or relapsing disease or for the treatment of severe diffuse alveolar hemorrhage (42). The largest trial investigating role of PLEX, the MEPEX trial (High-Dosage Methylprednisolone or Plasma Exchange as Adjunctive Therapy for Severe Renal Vasculitis) (59) showed an increased rate of renal recovery in AAV patients presenting with renal failure when compared with intravenous methylprednisolone. However, this trial enrolled patients who were dialysis dependent or nearing end stage renal disease and it was unable to identify role of PLEX as an adjunctive to conventional therapies. Long-term follow-up of the same cohort failed to identify sustained benefit in PLEX group (60). PEXIVAS is an international study which enrolled patients with AAV with severe renal vasculitis and/or diffuse alveolar hemorrhage. This study aims to determine if the adjunctive plasma exchange with two oral glucocorticoid regimens (standard- and reduced-dose GC) with standard remission induction immunosuppression is effective in reducing death and end-stage renal disease (61).

As mentioned above, clinical trials have not been conducted in pediatric GPA. The data from ARChiVe cohort highlights use of GC and CYC pulses most commonly used as a part of induction therapy (both for GPA and MPA) followed most frequently with methotrexate as a maintenance regime (6). However, most pediatric patients are now treated according to the adult recommendations (42, 43). Many North American centers now prefers RTX as first-line remission induction therapy in children with severe GPA or MPA as it has low toxicity profile. PLEX should be considered in children with severe pulmonary hemorrhage or rapidly progressive renal disease responding inappropriately to GC and CYC or RTX.

## Emerging therapy in ANCA vasculitis

The better understanding of the pathogenesis of ANCA vasculitis from *in vitro* and animal studies have helped us to identify the targeted therapies focusing on components of innate and adaptive immune system.

Belimumab (BEL) was investigated in a phase III, multicenter randomized double-blind trial evaluating its role the maintenance of remission in GPA and MPA in combination with AZA (BREVAS: NCT01663623). In BREVAS trial, addition of BEL to maintenance therapy with AZA did not reduce the risk of relapse. Fewer relapses of vasculitis was identified in RTX induced patients compared with placebo (62).

Abatacept, a fusion protein co-stimulatory T cell blocker was evaluated in an open labeled study in non-severe relapsing GPA. Almost 90% patient had disease improvement and >70% patients could discontinue prednisone. The study was limited by a small sample size, continuing background DMARDS and prednisone early during the study (63). Abatacept is currently being investigated in a multicenter, phase III, double-blind, placebo-controlled trial in the treatment of non-severe AAV (ABROGATE: NCT02108860).

The role of blocking complement component/receptor has been explored. Avacopan (CCX168/ selective C5a receptor inhibitor) was investigated in a phase II randomized, placebo-controlled trial (CLEAR: NCT01363388). Results from this study indicate both treatment groups receiving CCX168 were non-inferior to the standard induction and high-dose prednisone. These results highlight the importance of C5a as an important inflammatory mediator in AAV and can be used in future as an alternative to the use of oral glucocorticoids (64). The efficacy of Avacopan is now being evaluated in a larger phase III randomized, double-blind, active-controlled study (ADVOCATE: NCT02994927). Eculizumab is a long-acting humanized monoclonal antibody targeted against complement C5 and inhibits the deployment of the terminal complement system including the formation of membrane attack complex. It has been used successfully in a case report as an add-on treatment with an excellent clinical response with complete recovery of renal function (65).

Gusperimus (15-deoxyspergualin) inhibits mainly T-cell maturation and cytotoxic T-cell proliferation. It was used in a small open labeled trial with high response rates in refractory GPA (66). Clinical trial assessing the efficacy of gusperimus compared to conventional treatment was prematurely terminated (SPARROW: NCT NCT01446211). IL-6 is expressed and produced at sites of active vasculitis and levels are increased in patients with AAV (67). Case reports of successful treatment with Tocilizumab have been published in literature (67, 68). Clinical trial with TCZ and AAV is currently being considered (69).

## Outcome/prognosis

The mortality rates reported in pediatric series are low. The French registry reported a mortality of 10% (70) as compared to none in a single center series reported by Noone et al. (71) and James et al. (33) in pediatric patients with predominant AAV associated glomerulonephritis and GPA respectively. The French vasculitis study group registry reported an increased incidence of ischemic abdominal pain and damage involving the upper respiratory tract. Childhood AAV relapse rates were also reported much higher requiring longer maintenance therapy compared to adult AAV (72). Important morbidity associated with AAV in pediatric patients includes nasal septal perforation with saddle nose deformity, chronic sinusitis, osteoporosis, chronic kidney disease, end stage renal disease, cystitis, infertility, and avascular necrosis.

Recently published early treatment outcome data, reported under 50% of patients achieving remission at 12 months and 61% with inactive disease at 12 months. Improvement of PVAS score of at least 50% from time of diagnosis to post-induction was seen in 92% of patients. Vasculitis associated disease damage with PVDI scores ≥1 was identified in more than 60% of the patient cohort early in their disease course (73).

## Conclusion

AAV are rare life threatening severe illnesses of childhood associated with significant organ damage. The pathogenesis of AAV is unclear but both the innate and adaptive arms of the immune system play a role in the disease causation. The collaborative efforts among pediatric rheumatologist have helped in recognizing common clinical features and treatment choices in these rare AAV illness. The treatment of AAV is extrapolated from the adult studies with cyclophosphamide and glucocorticoids continuing to remain the main choices for induction regime, and rituximab gaining increasing popularity recently. Larger collaborative efforts to conduct international multicenter pediatric clinical trials are required to determine the efficacy of the existing treatment, to devise validated disease activity and damage indices and to better define the long-term outcome of pediatric AAV.

## Author contributions

MJ reviewed the literature and prepared the manuscript. RL reviewed the literature and prepared the manuscript.

### Conflict of interest statement

The authors declare that the research was conducted in the absence of any commercial or financial relationships that could be construed as a potential conflict of interest.
